# Reappraisal of the Role of Alkaline Phosphatase in Hepatocellular Carcinoma

**DOI:** 10.3390/jpm12040518

**Published:** 2022-03-23

**Authors:** Chun-Wei Huang, Tsung-Han Wu, Heng-Yuan Hsu, Kuang-Tse Pan, Chao-Wei Lee, Sio-Wai Chong, Song-Fong Huang, Sey-En Lin, Ming-Chin Yu, Shen-Ming Chen

**Affiliations:** 1 Division of General Surgery, Department of Surgery, New Taipei Municipal Tucheng Hospital (by Chang Gung Medical Foundation, and Chang Gung University and Shen-Ming Chen), New Taipei 23652, Taiwan; hugo7485@hotmail.com (C.-W.H.); smallredshoe@gmail.com (H.-Y.H.); b9602095@cgmh.org.tw (S.-W.C.); b910205@cgmh.org.tw (S.-F.H.); 2 Department of Surgery, Chang Gung Memorial Hospital, Linkou and Chang-Gung University, Taoyuan 33305, Taiwan; wutsunghan@gmail.com (T.-H.W.); alanchaoweilee@hotmail.com (C.-W.L.); 3 Department of Medical Imaging and Intervention, Linkou Chang Gung Memorial Hospital, Taoyuan 33305, Taiwan; pan0803@cgmh.org.tw; 4 Department of Pathology, New Taipei Municipal Tucheng Hospital, New Taipei 23652, Taiwan; linse@cgmh.org.tw; 5 Department of Chemical Engineering and Biotechnology, National Taipei University of Technology, Taipei 106, Taiwan

**Keywords:** hepatocellular carcinoma, alkaline phosphatase, liver regeneration

## Abstract

Background: Alkaline phosphatase (ALP) is a marker of liver function and is associated with biliary tract disease. It was reported as a prognostic factor for hepatocellular carcinoma (HCC). The genetic expression in tumor-tissue microarrays and the perioperative serologic changes in ALP have never been studied for their correlation with HCC prognosis. Methods: The genetic expression of ALP isoforms (placental (ALPP), intestinal (ALPI) and bone/kidney/liver (ALPL)) was analyzed in tumor and non-cancerous areas in 38 patients with HCC after partial hepatectomy. The perioperative change in ALP was further analyzed in a cohort containing 525 patients with HCC to correlate it with oncologic outcomes. A total of 43 HCC patients were enrolled for a volumetry study after major and minor hepatectomy. Results: The genetic expression of the bone/kidney/liver isoform was specifically and significantly higher in non-cancerous areas than in tumors. Patients with HCC with a higher ALP (>81 U/dL) had significantly more major hepatectomies, vascular invasion, and recurrence. Cox regression analysis showed that gender, major hepatectomies, the presence of satellite lesions, higher grades (III or IV) and perioperative changes in liver function tests were independent prognostic factors for recurrence-free survival, and a postoperative increase in the ALP ratio at postoperative day (POD) 7 vs. POD 0 > 1.46 should be emphasized. A liver regeneration rate more than 1.8 and correlation analysis revealed that the ALP level at POD 7 and 30 was significantly higher and correlated with remnant liver growth. Conclusions: This study demonstrated that the perioperative ALP change was an independent prognostic factor for HCC after partial hepatectomies, and the elevation of ALP represented a functional biomarker for the liver but not an HCC biomarker. The higher regeneration capacity was possibly associated with the elevation of ALP after operation.

## 1. Introduction

Hepatocellular carcinoma (HCC) is one of the most common cancers and is a leading cause of cancer death worldwide [1]. Partial hepatectomy, ablation therapy, and liver transplantation are considered curative treatments for HCC, but the high probability of recurrence has led to unsatisfactory outcomes, and multimodality treatment is now more important than before. Recently, preoperative alkaline phosphatase (ALP) has been identified as an independent prognostic factor for recurrence in patients with HCC [2]. The elevation of serum protein induced by vitamin K antagonist-II (PIVKA-II) and ALP are two independent factors reported by a Korean group [3]. ALP has also been incorporated into a predicted series formula to predict HCC recurrence after partial hepatectomies, such as the Chinese university prognostic index (CUPI), albumin/alkaline phosphatase ratio (AAPR), ALP plus gamma-glutamyl transpeptidase (GGT)/lymphocyte ratio (AGLR), and GGT-to-ALP ratio [4,5,6], suggesting that ALP is important in different HCC studies.

ALP is confined to the cell surface and releases inorganic phosphate in different kinds of tissue [7]. There are at least four isoforms according to tissue specificity: placental, intestinal, liver/bone/kidney, and germ cell. The physiological function is obscure, except in relation to bone skeletal mineralization [8]. ALP is elevated in biliary obstruction and liver parenchymal disease, but the biological function and impact of ALP remain an issue in disease and neoplastic conditions [9]. Hierarchical clustering analysis for 1685 HCCs shows that ALP (>82 IU/L) is associated with the elevation of aspartate aminotransferase (AST; >43 U/L), alanine aminotransferase (ALT; >42 U/L), and the bilirubin level and the presence of cirrhosis, whereas the tumor status is associated with vascular invasion, satellite lesions, and a lack of tumor encapsulation [2]. ALP is elevated at postoperative day (POD) 7 for living-donor hepatectomies and is also used to predict liver fibrosis [10,11,12]. ALP is more associated with liver non-cancerous parenchyma change than liver HCC biomarkers.

Preoperative ALP has been incorporated into different formulas predicting patient outcome. However, there are few reports on the perioperative change in ALP in patients with HCC. In this study, we analyzed the biological impact of ALP from the genetic expression perspective and identified the dynamic change in ALP postoperatively. Analysis of the association of the dynamic change in ALP with oncologic outcomes and the change in the elevation of ALP with liver regeneration was performed to explore the clinical significance of ALP in HCC after operation.

## 2. Materials and Methods

**Figure 1:**
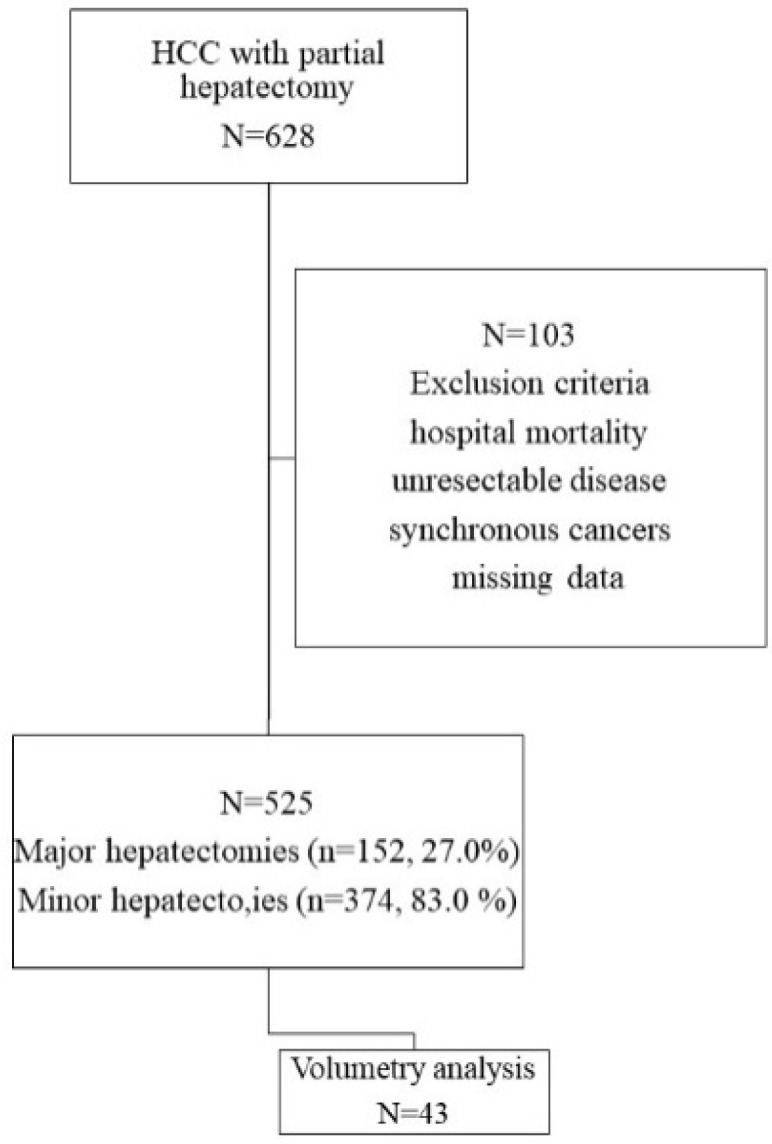
The study design for the role of alkaline phosphatase in HCC. Of 628 HCC patients reviewed, 525 were eligible for analysis and 43 of them were further analyzed in volumetry analysis.

Of 628 HCC cases with intended curative treatment from 2012 till 2018, 103 patients with hospital mortality, unresectable disease, synchronous cancers or missing data were excluded. A total of 525 patients with partial hepatectomy were enrolled for the analysis of perioperative parameters for long-term outcomes. The study endpoint was February 2021. The median follow-up time was 42.6 months, and the mean was 45.5 ± 23.3 months (IQR, 25th–75th percentiles, 28.6–66.0). The tumor staging was based on the 8th edition of the American Joint Committee on Cancer TNM staging system for HCC [13]. This retrospective cohort study was approved by the Institutional Review Board (IRB 201701002B0) of Chang Gung Memorial Hospital, Linkou Branch, and informed consent was waived. The study design is shown in the flow chart (Figure 1).

### 2.1. Liver Regeneration Rate (LRR) Analysis

The LRR was calculated from the ratio of the regenerated liver volume at POD 28 versus the estimated preserved liver volume at POD 0 using computed-tomography-based volumetry. The patients were dichotomized into two groups according to major and minor hepatectomies, by three anatomic segments [2].

### 2.2. Transcriptional Gene Expression

Another cohort consisting of 38 patients with stage I/II HCC were enrolled to analyze transcriptional gene expression (IRB No. 201201186B0). Total RNA was extracted using TRIzol™, as recommended by the manufacturer, followed by RNA cleanup using the MinElute Kit (Thermo Fisher Scientific Inc., Waltham, MA, USA). RNA labeling, hybridization, washes, and processing were performed by the Genomic Medicine Research Core Laboratory of Chang Gung Memorial Hospital using an Affymetrix GeneChip™ Human Genome U133 Plus 2.0 Array (Thermo Fisher Scientific Inc., Waltham, MA, USA). To filter the lower-variance genes, a standard deviation >0.5 was used to filter 6522 probe sets from the original 22,215. The genes differentially expressed between cancerous and non-cancerous tissues were identified using paired *t*-tests, and the *p* values for gene expression were calculated as previously described [14,15].

### 2.3. Statistical Analysis

Statistical analysis was performed using IBM SPSS Statistics v21 (IBM Corp., Somers, NY, USA). Fisher’s exact test and Pearson’s χ^2^ test were used to analyze categorical data. Student’s *t*-test was used to analyze quantitative variables. The serial change was compared and expressed with paired *t*-tests. The continuous data were analyzed with the Youden index (or Youden’s J statistic) to determine the best cut-off value for dichotomization for further work. The disease-free survival was calculated from the date of surgery to the date of the first documented clinical disease recurrence. Cases with surgical mortality, defined as death within one month of surgery, were excluded from survival analyses. Kaplan–Meier analysis was used to determine the recurrence-free survival. The log-rank test and Cox regression multivariate analysis were used to determine the prognostic significance of clinicopathological variables. Statistical significance was defined as *p* < 0.05.

## 3. Results

### 3.1. Higher Expression of Liver-Specific ALP in Non-Cancerous Areas of HCC

**Figure 2:**
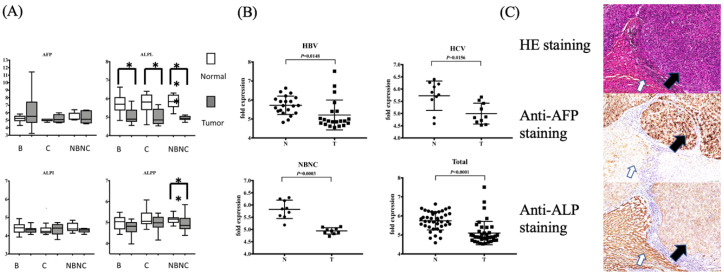
Higher expression of liver-specific alkaline phosphatase (ALP) in hepatocellular carcinoma (HCC) non-cancerous areas. (**A**) Analysis of genetic expression in HCC non-cancerous (N) and tumor (T) areas shows significant elevation of ALP. (**B**) Higher genetic expression of ALP is consistent among different patient etiologic factors. (**C**) Representative immunohistochemical staining of α-fetoprotein (AFP) and ALP in HCC. Stronger staining is noted at non-cancerous tumor area for ALP and tumor area for AFP (200 × X magnification). (* *p* < 0.05).

The genetic expression of HCC was analyzed using the Affymetrix GeneChip human genome U133 plus 2.0 array [2,16]. A total of 38 patients with HCC were enrolled and analyzed for genetic expression and the levels of α-fetoprotein (AFP), and placental, intestinal and bone/kidney/liver isoforms of ALP (ALPP, ALPI and ALPL, respectively). AFP was commonly the tumor biomarker that was expressed more highly in tumors (*p* = 0.038). Although ALP commonly existed in different organ systems, the expression of the bone/kidney/liver isoform was specifically and significantly higher in non-cancerous areas than in tumors (Figure 2A, *p* < 0.001). The importance of higher ALP genetic expression was consistent among different etiologic patient factors; however, there was no remarkable difference in the expression of intestinal or placental isoforms (Figure 2B). The immunohistochemical staining of ALP showed higher expression in non-cancerous areas than in the tumor part, but that of AFP showed higher expression in the tumor part (Figure 2C). Therefore, ALP was highly expressed in non-cancerous areas, but not in tumors.

### 3.2. Perioperative ALP Change was an Independent Factor for Oncologic Outcome in HCC

**Figure 3:**
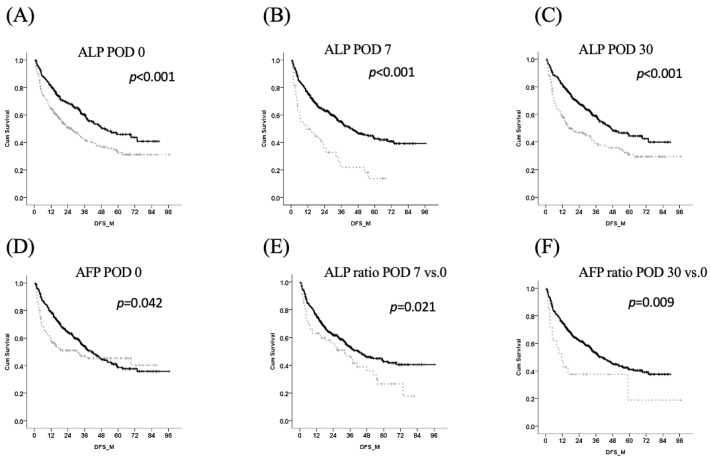
The analysis of recurrence-free survival. Kaplan–Meier survival curve for AFP and ALP in HCC in the perioperative period. (**A**), (**B**) and (**C**) Higher ALP in perioperative period at POD 0, 7 and 30 represented significant risk for HCC recurrence (*p* < 0.001). (**D**) AFP more than 200 ng/mL is a risk factor for recurrence, too. (**E**) and (**F**) ALP ratio > 1.46 (POD 7 vs. POD 0) and AFP ratio (POD 30 vs. POD 0) > 1.519 were independent factors for recurrence-free survival. Abbreviations: alkaline phosphatase (ALP) and α-fetoprotein (AFP).

A total of 525 patients with HCC were enrolled for the analysis of the perioperative change in ALP. The demographic data are shown in Table 1. There were 228 patients with ALP levels higher than 81 U/L and 297 patients with ALP levels less than 81 U/L (122.4 ± 78.3 and 63.1 ± 11.1 U/L, respectively, *p* < 0.001). The tumor size, vascular invasion, AST, ALT, and albumin levels, but not AFP levels, were significantly different between both groups (*p* < 0.001). Patients with HCC with higher ALP levels have significantly more major hepatectomies and recurrence, which is in agreement with our previous study [2]. The perioperative change was determined, and Cox regression analysis showed that gender, major hepatectomies, the presence of satellite lesions, higher grades (III or IV), AST > 59 U/L at POD 0, ALP > 136 U/L at POD 2, ALT > 72 U/L at POD 7, AST < 44 U/L at POD 30, ALT > 162 U/L at POD 30, albumin < 3.75 mg/dL at POD 30, AFP > 166.1 ng/mL at POD 30, an AFP ratio (POD 30 vs. POD 0) > 1.519, and an ALP ratio > 1.46 (POD 7 vs. POD 0) were independent factors for recurrence-free survival (Table 2 and Figure 3). The perioperative changes in liver function tests, including those for AST, ALT, ALP and albumin, are independent prognostic factors for HCC after partial hepatectomies.

### 3.3. Analysis of Liver-Function Parameters between Major and Minor Hepatectomies for HCC

**Figure 4:**
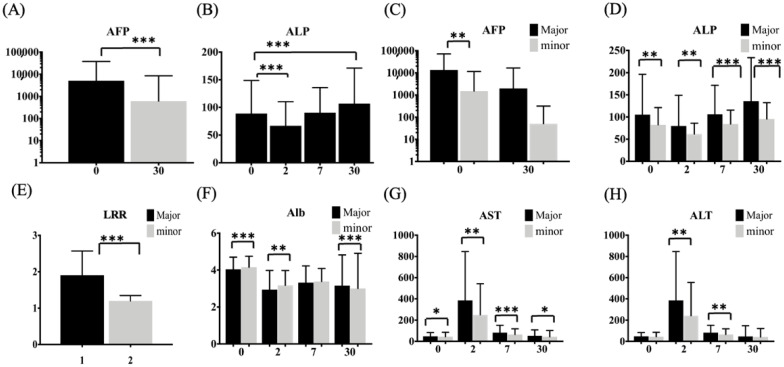
Change in AFP and liver-function tests in the perioperative period between major and minor hepatectomies for HCC. (**A**) and (**B**) AFP was significantly decreased after operation, but ALP was significantly decreased at POD 2 and increased at POD 30 according to paired t-test. Patients with HCC had dynamic liver functional changes. (**C**) AFP showed significantly different between major and minor groups on POD 0. AST (**G**), ALT (**H**), ALP (**D**), albumin (**F**) and AFP showed significant differences in major and minor hepatectomies. The liver regeneration ratio (**E**) was calculated with postoperative liver volume/estimated preserved liver volume. The regeneration ratios were 1.91 ± 0.66 and 1.20 ± 0.15, respectively (*p* < 0.001). However, there was a dynamic change after partial hepatectomies, but ALP was significantly increased in both groups at POD 7 and POD 30. Abbreviations: aspartate aminotransferase (AST), alanine aminotransferase (ALT), alkaline phosphatase (ALP), and albumin (Alb). * Statistical significance (* *p* < 0.05, ** *p* < 0.01, *** *p* < 0.001).

For all the patients, the AFP was significantly decreased after hepatectomies, but the ALP significantly decreased at POD 2 and increased at POD 30 in all the patients (*p* < 0.001) (Figure 4A,B). A significantly inverse presentation between the perioperative changes in AFP and ALP was noted. HCC patients with major hepatectomies showed higher perioperative serologic changes and had higher LRRs, compared with the minor group (Figure 4D–H). The AST and ALT levels, representing the degree of liver damage, rose to the highest levels at POD 2, but the ALP level was decreased at POD 2 and increased at POD 7 and POD 30. Therefore, ALP, released from the nontumor part after resection, represented a dynamic change in the liver remnant.

**Figure 5:**
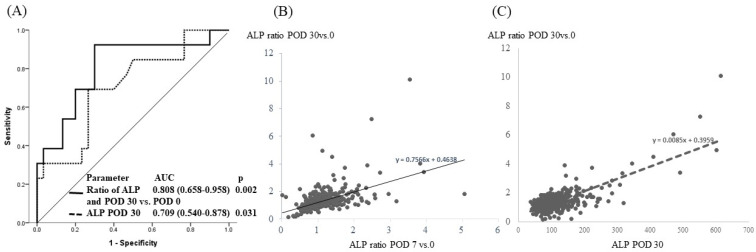
The perioperative ALP change and liver regeneration. (**A**) Total of 43 patients with volumetry analysis; AUROCs for liver regeneration rate >1.8 of ALP ratio (POD 30 vs. POD 0) and ALP at POD 30 were 0.808 (0.658–0.958) and 0.709 (0.540–0.878) (*p* = 0.002 and 0.031, respectively). (**B**) The increase in ALP after partial hepatectomies. The ALP ratio of POD 7 vs. 0 was significantly correlated with that of ALP POD 30 vs. 0 (Pearson correlation = 0.757, *p* < 0.001). (**C**) The ratio of ALP at POD 30 vs. 0 was significantly correlated with that of ALP at POD 30 (Pearson correlation = 0.919, *p* < 0.001).

Of the 525 patients, 43 were further analyzed for liver volume analysis. All the patients had Child-Pugh A status. The total liver volume before operation was 1492.1 ± 848.1 cm^3^, and the estimated preserved liver volume was 680.0 ± 251.8 cm^3^. The liver regeneration rate (LRR) was 1.66 ± 0.64. There was a significant volume increase in the major hepatectomy group (Figure 4E). The patients were dichotomized into lower and higher groups according to an LRR of 1.8, and there was a marginally significant difference in the ALP at POD 30 and the ALP ratio at POD 30 vs. 0 between the higher and lower LRR groups (Table 3, *p* = 0.074 and 0.082, respectively). Furthermore, the area under the receiver operating characteristic (AUROC) curve of the ALP ratio (POD 30 vs. POD 0) and ALP at POD 30 was significantly higher than that for the other liver-function tests (Figure 5A; AUC = 0.808 (0.658–0.958) and 0.709 (0.540–0.878), *p* = 0.002 and 0.031, respectively). The increases in ALP after partial hepatectomies were significantly correlated for the ALP ratio at POD 7 vs. 0 and POD 30 vs. 0 (Pearson correlation = 0.757, *p* < 0.001) and for the ALP at POD 30 and ratio of ALP at POD 30 vs. 0 (Pearson correlation = 0.919, *p* < 0.001). A dynamically physiological change in the liver-function tests was noted, especially when LRR > 1.8 (Figure 5B,C).

## 4. Discussion

ALP is a common serologic test for liver function, especially for cholestasis, and it is also an indicator for liver regeneration, when living donors undertake donor hepatectomies [10]. The ALP liver isoform was gradually increased and reached a peak on POD 14 in a high-liver-regeneration group (regeneration rate over 1.5) [11]. ALP is elevated in rats with liver injury and regresses after treatment with nitric-oxide-synthesis inhibitors to improve liver function and attenuate liver cirrhosis [17]. In our study, the dynamic change in ALP was shown to have a strong correlation with liver regeneration. The ALP ratio (POD 30 vs. 0) and ALP at POD 30 showed strong differences between the higher and lower LRR groups. This is the first study for the perioperative dynamic change in ALP and its association with HCC and liver regeneration.

The bone/kidney/liver isoform specifically showed serologic change after partial hepatectomy. This was demonstrated by microarray analysis in these experiments, in patients with either hepatitis B or C viral infection and without infection. ALP is a prognostic factor in the CUPI staging system and our early studies, but there are few reports on the serologic data transition in perioperative periods [18]. ALP levels were elevated after surgery for 7–14 days for major hepatectomies and returned to normal levels one month later. The dynamic transitional change reflected liver-function recovery and a regenerated liver volume after surgery.

Early AFP reduction is one of the most common biomarkers and predictors in HCC treatment, compared to the modified response evaluation criteria in solid tumors, but this is not consistent in various cohorts [19,20]. It is also a good marker for ramucirumab treatment in Registration, Evaluation, Authorization and Restriction of Chemicals trials [21]. The pretreatment levels and 50% reduction after treatment for other biomarkers, such as AFP-L3 and PIVKA-II, are also reported [22,23,24]. However, ALP is seldom reported. In this cohort, more than 95% of the patients developed AFP level reductions one month after surgery, and a postoperative increase in the AFP ratio over 1.52 is associated with recurrence. The ALP ratio (POD 7 vs. POD 01.462) was an independent factor for disease-free survival. However, the ALP levels were significantly higher than the levels before surgery. Therefore, it was not a tumor-associated biomarker but reflected the change in the microenvironment in non-cancerous areas.

This study reported the perioperative change in ALP in HCC outcomes after partial hepatectomies. There were some limitations here; the data were from a retrospective cohort study, and a randomized prospective study will have more significance for clinical science; the perioperative change in ALP in terms of the biological change in HCC needs translation research to prove it, and the impact of liver regeneration in the HCC outcome after curative treatment was not fully analyzed. Further studies should be considered to decipher the association of ALP and liver-regeneration activity

## 5. Conclusions

In conclusion, this study demonstrated that perioperative ALP change was an independent prognostic factor for HCC after partial hepatectomies, and the elevation of ALP represented a functional biomarker for the liver but not an HCC biomarker. The higher regeneration capacity was possibly associated with the elevation of ALP after operation.

## Figures and Tables

**Table 1:** Demographic data of 525 HCC patients; comparison of higher and lower ALP level.

Variables	All	ALP ≤ 81 IU/L (*n* = 297)	ALP > 81 IU/L (*n* = 228)	*p*
Age	61.4 ± 11.8	60.2 ± 11.2	62.9 ± 11.5	0.007 **
Gender (male)	410 (78.1)	246 (82.8)	164 (71.9)	0.004 **
Comorbidity (yes)	322 (61.3)	176 (59.3)	146 (64.0)	0.279
HBV positive	311 (59.2)	189 (63.6)	122 (53.5)	0.020 *
HCV positive	147 (28.0)	70 (23.6)	77 (33.8)	0.011 *
ICG R15	10.2 ± 8.0	9.5 ± 8.2	11.2 ± 7.5	0.026 *
Major hepatectomy	152 (29.0)	72 (24.0)	78 (34.2)	0.020 *
Complication (yes)	33 (6.3)	14 (4.7)	19 (8.4)	0.088
ALP (IU/L)	88.9 ± 59.9	63.1 ± 11.1	122.4 ± 78.3	<0.001 ***
AST (IU/L)	45.3 ± 31.8	37.6 ± 20.7	55.3 ± 40.0	<0.001 ***
ALT (IU/L)	45.1 ± 40.0	37.5 ± 28.4	55.0 ± 49.8	<0.001 ***
BIL (mg/dL)	0.7 ± 0.3	0.7 ± 0.3	0.6 ± 0.3	0.054
ALB (g/dL)	4.2 ± 0.4	4.3 ± 0.4	4.1 ± 0.4	<0.001 ***
AFP (ng/mL)	4987.5 ± 32,890.0	2920.9 ± 19,665.6	7679.6 ± 44,506.7	0.133
AFP (>400 ng/mL)	126 (24.0)	65 (21.9)	61 (26.8)	0.195
Cirrhosis	252 (48.1)	134 (45.1)	118 (52.0)	0.119
Satellite lesion	73 (13.9)	41 (13.8)	32 (14.0)	1.000
Vascular invasion				
No	324 (61.7)	200 (67.3)	124 (54.4)	
Microscopic	160 (30.5)	79 (26.6)	81 (35.5)	
Gross	41 (7.8)	18 (6.1)	23 (10.1)	0.008 **
Margin < 1 cm	115 (21.9)	68 (22.9)	47 (20.6)	0.595
Tumor size > 5 cm	154 (29.3)	67 (22.6)	87 (38.2)	<0.001 ***
Tumor size (cm)	4.7 ± 3.5	4.0 ± 2.6	5.6 ± 4.2	<0.001 ***
Rupture	42 (8.0)	18 (6.1)	24 (10.5)	0.074
Grade III, IV	225 (43.4)	127 (43.1)	98 (43.8)	0.924
AJCC 8 staging				0.074
III	238 (45.3)	122 (41.1)	116 (50.9)	
II	212 (40.4)	131 (44.1)	81 (35.5)	
I	75 (13.8)	44 (14.8)	31 (13.6)	
Recurrence	253 (48.2)	124 (41.8)	129 (56.6)	0.001 **

* Statistical significance (*p* < 0.05, ** *p* < 0.01, *** *p* < 0.001); HBV: hepatitis B virus; HCV: hepatitis C virus; AST: aspartate aminotransferase; ALT: alanine aminotransferase; ALP: alkaline phosphatase; BIL: bilirubin; ALB: albumin; AFP: alpha-fetoprotein; AJCC 8 staging: the 8th edition of American Joint Committee on Cancer TNM staging system.

**Table 2:** Clinicopathologic data and sequential change in 525 HCC patients in univariate and multivariate regression analysis.

Variable	Univariate Analysis			Multivariate Analysis		
	HR	95% CI	*p* Value	HR	95% CI	*p* Value
Age (years), >74 (12.0%) vs. ≤74 (88.0%)	0.765	0.502–1.165	0.212			
Gender (M/F), F (21.9%) vs. M (78.1%)	0.694	0.501–0.961	0.028 *	0.508	0.307–0.840	0.008 **
Complication, yes (6.3%) vs. no (93.7%)	1.561	0.953–2.557	0.077			
Comorbidity, yes (61.3%) vs. no (38.7%)	1.110	0.858–1.436	0.427			
HBV, yes (59.2%) vs. no (40.8%)	1.086	0.846–1.396	0.516			
HCV, yes (28.0%) vs. no (72.0%)	0.857	0.658–1.116	0.251			
Hepatectomies, major (29.0%) vs. minor (71.0%)	1.748	1.342–2.272	<0.001 ***	1.695	1.024–2.806	0.040 *
Blood loss (500 mL), more (29.9%) vs. less (70.1%)	1.529	1.118–1.980	0.001 ***	1.487	1.004–2.202	0.048 *
Tumor size (5 cm), >5.0 (29.3%) vs. ≤5.0 (70.7%)	2.216	1.716–2.863	0.001 ***	1.044	0.628–1.733	0.869
Satellite lesions (%), yes (13.9%) vs. No (86.1%)	2.250	1.657–3.055	0.001 ***	1.793	1.070–3.002	0.026 *
Vascular invasion (%), no (61.7%) vs. microscopic (30.5%) vs. thrombus (7.8%)	1.728	1.446–2.066	0.001 ***	1.331	0.952–1.861	0.095
Grading I/II/III, IV (%), III, IV (43.4%) vs. I, II (56.6%)	1.376	1.072–1.766	0.012 *	1.557	1.077–2.251	0.019 *
Margin <1cm (%), ≤1 cm (78.1%) vs. >1 cm (21.9%)	1.321	0.969–1.800	0.078	1.106	0.733–1.669	0.632
Cirrhosis, yes (51.9%) vs. No (48.1%)	0.804	0.628–1.031	0.085			
Rupture, yes (8.0%) vs. No (92.0%)	1.988	1.340–2.950	0.001 ***	0.818	0.447–1.495	0.514
Encapsulation, yes (86.5%) vs. No (13.5%)	1.154	0.808–1.649	0.431			
AJCC 8th Stage ^a^ III (45.3%) vs. II (40.4%) vs. I (14.3)	1.868	1.535–2.274	<0.001 ***	1.235	0.871–1.750	0.236
AST (59.8 IU/L) POD 0, high (20.2%) vs. low (79.8%)	1.732	1.306–2.298	<0.001 ***	1.996	1.121–3.554	0.019 *
ALT (76 IU/L) POD 0, high (11.2%) vs. low (88.8%)	1.502	1.046–2.158	0.028 *	0.839	0.415–1.696	0.625
Bilirubin (0.4 mg/dL) POD 0, high (77.1%) vs. low (22.9%)	1.243	0.919–1.680	0.157			
ALP (81 IU/L) POD 0, high (43.4%) vs. low (56.6%)	1.640	1.281–2.099	<0.001 ***	1.410	0.904–2.199	0.130
ALB (3.94 g/dL) POD 0, high (71.9%) vs. low (28.1%)	0.700	0.538–0.911	0.008 **	1.187	0.773–1.823	0.434
AFP (200 ng/mL) POD 0, high (24.0%) vs. low (76.0%)	1.340	1.010–1.777	0.042 *	1.098	0.697–1.728	0.687
AST (96 IU/L) POD 2, high (72.0%) vs. low (28.0%)	1.302	0.982–1.727	0.067			
ALT (775 IU/L) POD 2, high (7.1%) vs. low (92.9%)	0.984	0.722–1.341	0.919			
Bilirubin (0.7 mg/dL) POD 2, high (84.3%) vs. low (15.7%)	1.428	0.969–2.1039	0.071			
ALP (136 IU/L) POD 2, high (3.3%) vs. low (96.7%)	3.983	2.355–6.739	<0.001 ***	2.442	1.120–5.321	0.025 *
ALB (3.45 g/dL) POD 2, high (37.2%) vs. low (62.8%)	0.545	0.410–0.724	<0.001 ***	0.814	0.544–1.217	0.316
AST (75IU/L) POD 7, high (9.4%) vs. low (90.6%)	1.769	1.201–2.606	0.004 ***	1.289	0.679–2.446	0.438
ALT (72 IU/L) POD 7, high (33.7%) vs. low (66.3%)	0.739	0.561–0.973	0.031 *	0.568	0.366–0.881	0.012 *
ALP (141 IU/L) POD 7, high (8.4%) vs. low (91.6%)	2.351	1.621–3.409	<0.001 ***	1.281	0.665–2.467	0.459
Bilirubin (0.8 mg/dL) POD 7, high (33.0%) vs. low (67.0)	1.458	1.130–1.881	0.004 **	1.119	0.756–1.657	0.574
ALB (3.69 g/dL) POD 7, high (27.5%) vs. low (72.5%)	0.741	0.550–0.999	0.049 *	0.710	0.470–1.074	0.105
AST (44 IU/L) POD 30, high (31.6%) vs. low (68.4%)	1.461	1.129–1.891	0.004 **	0.560	0.363–0.864	0.009 **
ALT (162 IU/L) POD 30, high (2.3%) vs. low (97.7%)	2.989	1.533–5.827	0.001 ***	3.757	1.432–9.857	0.007 **
ALP (107 IU/L) POD 30, high (32.4%) vs. low (67.6%)	1.778	1.382–2.286	<0.001 ***	1.254	0.800–1.965	0.324
Bilirubin (0.9 mg/dL), high (13.1%) vs. low (86.9%)	1.541	1.085–2.187	0.016 *	1.156	0.670–1.995	0.601
ALB (3.75 g/dL) POD 30, high (81.7%) vs. low (18.3%)	0.532	0.377–0.749	<0.001 ***	0.566	0.359–0.892	0.014 *
AFP (166.1 ng/mL) POD 30, high (5.7%) vs. low (94.3%)	5.992	3.967–9.051	<0.001 ***	3.620	1.669–7.854	0.001 ***
ALP (0.696) POD 2 vs. 0, high (70.9%) vs. low (29.1%)	0.731	0.563–0.951	0.019 *	0.786	0.512–1.208	0.272
ALP (1.462) POD 7 vs. 0, high (13.1%) vs. low (86.9%)	1.479	1.062–2.059	0.021 *	2.082	1.158–3.743	0.014 *
ALP (0.980) POD 30 vs. 0, high (79.6%) vs. low (20.4%)	0.751	0.563–1.002	0.052			
AFP (1.519) POD 30 vs. 0, high (4.0%) vs. low (96.0%)	2.046	1.193–3.511	0.009 **	2.369	1.051–5.337	0.037 *

* Statistical significance (* *p* < 0.05, ** *p* < 0.01, *** *p* < 0.001) in bold; HR, hazard ratio; 95% CI, 95% confidence interval of hazard ratio. Disease-free survival was calculated by univariate and multivariate Cox regression analysis. HBV: hepatitis B virus; HCV: hepatitis C virus; AST: aspartate aminotransferase; ALT: alanine aminotransferase; ALP: alkaline phosphatase; ALB: albumin; AFP: alpha-fetoprotein; AJCC 8 staging: the 8th edition of American Joint Committee on Cancer TNM staging system.

**Table 3:** Comparison of clinical parameters between lower and higher LRR after hepatectomies.

	LRR ≤ 1.8	LRR > 1.8	*p*
	*n* = 30	*n* = 13	
POD 0 (before hepatectomies)			
AST (IU/L)	51.1 ± 26.9	41.7 ± 20.0	0.262
ALT (IU/L)	45.9 ± 26.3	41.3 ± 37.3	0.649
ALP (IU/L)	85.1 ± 32.5	77.4 ± 19.8	0.432
BIL (mg/dL)	0.7 ± 0.3	0.7 ± 0.3	1.000
POD 30			
AST (IU/L)	50.3 ± 30.7	66.9 ± 42.1	0.154
ALT (IU/L)	46.0 ± 35.9	70.4 ± 60.5	0.107
ALP (IU/L)	102.3 ± 42.1	187.1 ± 153.8	0.074
BIL (mg/dL)	0.7 ± 0.3	0.7 ± 0.2 ^#^	0.903
Ratio of ALP (vs. POD 0)			
POD 2 vs. 0	0.80 ± 0.17	0.84 ± 0.15	0.485
POD 7 vs. 0	0.97 ± 0.29	1.38 ± 1.06	0.194
POD 30 vs.0	1.24 ± 0.39	2.02 ± 2.41	0.082

AST: aspartate aminotransferase; ALT: alanine aminotransferase; ALP: alkaline phosphatase; BIL: bilirubin; ALB: albumin; ^#^ One case developed biliary complications and was excluded.

## Data Availability

The data presented in this study are openly available in Figure 1 at doi:10.1245/s10434-011-1946-2, reference number [15].
